# The vertebrate Embryo Clock: Common players dancing to a different beat

**DOI:** 10.3389/fcell.2022.944016

**Published:** 2022-08-11

**Authors:** Gil Carraco, Ana P. Martins-Jesus, Raquel P. Andrade

**Affiliations:** ^1^ ABC-RI, Algarve Biomedical Center Research Institute, Faro, Portugal; ^2^ Faculdade de Medicina e Ciências Biomédicas (FMCB), Universidade do Algarve, Campus de Gambelas, Faro, Portugal; ^3^ Champalimaud Research Program, Champalimaud Center for the Unknown, Lisbon, Portugal

**Keywords:** temporal control, embryo clock, somitogenesis, negative feedback regulation, notch signalling, HES

## Abstract

Vertebrate embryo somitogenesis is the earliest morphological manifestation of the characteristic patterned structure of the adult axial skeleton. Pairs of somites flanking the neural tube are formed periodically during early development, and the molecular mechanisms in temporal control of this early patterning event have been thoroughly studied. The discovery of a molecular Embryo Clock (EC) underlying the periodicity of somite formation shed light on the importance of gene expression dynamics for pattern formation. The EC is now known to be present in all vertebrate organisms studied and this mechanism was also described in limb development and stem cell differentiation. An outstanding question, however, remains unanswered: what sets the different EC paces observed in different organisms and tissues? This review aims to summarize the available knowledge regarding the pace of the EC, its regulation and experimental manipulation and to expose new questions that might help shed light on what is still to unveil.

## 1 Highlights

• The vertebrate Embryo Clock oscillates with species-specific periodicity
• Embryo Clock periodicity is tissue-specific within the same organism
• A comprehensive concept of the Embryo Clock is presented

## 2 The somitogenesis Embryo Clock

Vertebrate embryo development comprises several processes that are highly regulated in time. One such process is somitogenesis, which is characterized by the periodic formation of metameric structures, the somites, along the anterior-to-posterior (A-P) axis of the early embryonic body. Somites are formed in pairs from the anterior-most portion of the presomitic mesoderm (PSM), on each side of the neural tube, and they are the first morphological manifestation of the characteristic segmented structure of the adult vertebrate axial skeleton. In fact, somites not only give rise to the axial skeleton and skeletal musculature, but also impose the segmented organization of the peripheral nervous system (Keynes and Stern, 1988). Most importantly to the subject of this review, somite pairs are formed sequentially, over time, while the embryonic body is elongating in an A-P direction. This is characteristic of all vertebrates, although the pace at which somites are formed varies among species (Table 1).

**TABLE 1:** Time of somite formation in different vertebrate organisms.

Organism	Time	References
Human	4—5 h	Müller and O’Rahilly, (1986)
Mouse	2–3 h	Tam, (1981)
Chicken	90 min	Palmeirim et al. (1997)
Quail	90 min	Packard, (1980)
Emu	100—110 min	Nagai et al. (2011)
Zebrafish	30 min	Kimmel et al. (1995)
Medaka	60 min	Iwamatsu, (2004)
*Xenopus*	40 min	Cooke and Zeeman (1976)
House snake	60 min	Gomez et al. (2008)
Corn snake	100 min	
Whiptail lizard	4 h	

In 1976, Cooke and Zeeman proposed a theoretical model that aimed to explain the formation of periodic structures during vertebrate development. In their *Clock and Wavefront* model (Cooke and Zeeman, 1976), the authors proposed the existence of two players: a molecular oscillator (*clock*), responsible for the rhythmic generation of a cell responsive state, and a maturation *wavefront*, moving slowly in an anterior-to-posterior direction. Exposure of a clock-induced cell population to the wavefront signal would promote a rapid change in cell properties, leading to the formation of a somite. Together, these two components would translate temporal information into a spatial pattern. According to this model, somite size and number are jointly determined by the period of the clock’s oscillations and the speed of the moving wavefront (Cooke and Zeeman, 1976; Oates et al., 2012). However, breakthroughs regarding the identity of the molecules comprising the *Clock* and the *Wavefront* were only made 20 years later.

The Embryo Clock (EC)—or the *developmental clock,* as it was first termed–arose from the discovery that the mRNA of chick *hairy1* (now termed *hes4*), a member of the Hairy Enhancer of Split (HES) transcription factor family, oscillated in the chicken embryo PSM with a 90 min periodicity, concomitant with the formation of a new pair of somites (Palmeirim et al., 1997). In their study, the authors first observed that chicken embryos with the same number of somites (i.e., within the same developmental stage) displayed very different patterns of *hairy1* expression, leading them to hypothesize that its expression could be cyclic. Indeed, by bisecting the embryo, and culturing one half for a given time while the other was immediately fixed, *hairy1* expression recapitulated after 90 min. Moreover, *hairy1* oscillations in the PSM were found to be an intrinsic property of the system, as they were maintained even when the PSM was sectioned in smaller pieces or isolated from the surrounding tissues (Palmeirim et al., 1997). Since then, many genes that display an oscillatory behaviour during somitogenesis have been identified in multiple organisms, evidencing that the EC underlying somitogenesis is a conserved mechanism among vertebrates (Krol et al., 2011).

The first evidence for a *Wavefront* in control of somite formation was provided soon after (Dubrulle et al., 2001 in chick; Sawada et al., 2001 in zebrafish). A gradient of *fgf8* mRNA (chick) and signalling activity (zebrafish) was described, with high levels at the embryo tail bud decreasing towards the anterior PSM. Local inhibition of FGF8 signalling in the anterior PSM resulted in longer somites, suggesting an instructive role for FGF signalling in positioning the somitic boundary (Dubrulle et al., 2001; Sawada et al., 2001). This was consistent with what was previously proposed for the *wavefront* activity (Cooke and Zeeman, 1976). Further studies elucidated that the chick *fgf8* mRNA gradient resulted from the production of stable mRNA transcripts in the tail bud region alone, that degraded over time as the embryo elongated posteriorly, leading to less mRNA levels in the anterior PSM relative to the posterior region (Dubrulle and Pourquié, 2004). Graded Wnt activity and an opposing, anterior-to-posterior gradient of retinoic acid signalling were further shown to have *wavefront* activity in defining somite boundary positioning (reviewed in Resende et al., 2014).

This review reunites and summarizes key findings on Embryo Clock operation over the last 25 years, since *hes4* oscillations in the chicken embryo were first described (Palmeirim et al., 1997). Originally termed “developmental clock” (Palmeirim et al., 1997), then “segmentation clock” (McGrew et al., 1998) and “somitogenesis clock” (Leimeister et al., 2000), herein we employ a more comprehensive concept of “Embryo Clock,” since oscillations in clock gene expression have been described in cells and developmental stages that are not associated with embryo somite formation (discussed below). We propose the term Embryo Clock to refer to the system of molecular oscillators operating in embryonic cells undergoing temporally controlled morphogenetic processes and/or cell fate specification. These genetic (or, in some cases, biochemical) oscillators exhibit periodic alterations (in contrast to stochastic pulses) that are maintained by negative feedback regulation. Due to the extensive and growing number of studies performed on the subject, we have focused our attention on the temporal dynamics of the EC. We aim to provide an overview of the main factors contributing to the exquisite temporal properties of this biological oscillator and anticipate this will be a useful roadmap for researchers interested in this increasingly exciting scientific field.

## 3 Gene expression oscillations

### 3.1 Embryo Clock genes in the PSM

**FIGURE 1:**
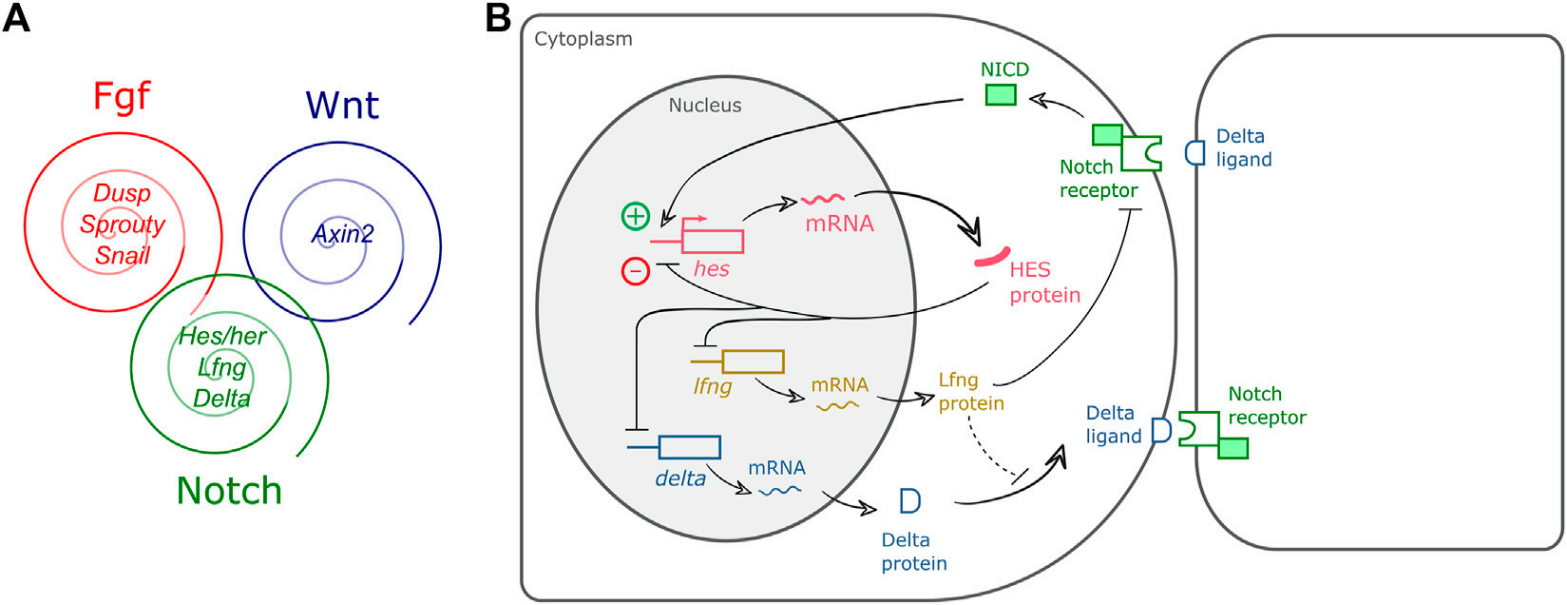
Embryo Clock (EC) gene expression oscillations. **(A)** The EC encompasses oscillatory genes belonging to the Fgf, Wnt and Notch signalling pathways (representative genes are indicated); **(B)** Negative feedback regulation of hairy-enhancer-of-split (HES) oscillations. In PSM cells, *hes* transcription is induced by pulses of intercellular Notch-Delta signalling, leading to HES protein production. HES protein enters the nucleus and represses its own promoter. HES protein and mRNA are rapidly degraded allowing for a new cycle of expression. HES also inhibits *delta* and *lfng* expression ensuring coupled oscillations in neighbour cells of the tissue. Dashed line represents a delay imposed on Delta integration in the cell membrane (Yoshioka-Kobayashi et al., 2020). NICD: Notch intracellular domain.

After the description of the first segmentation clock gene, *hairy1*, in the chicken embryo (Palmeirim et al., 1997), similar oscillatory patterns of expression were identified for other genes, and in multiple organisms (Table 2). The use of genome-wide approaches identified a wide range of genes with oscillatory gene expression during somitogenesis and evidenced that the embryo clock is an intricate oscillatory genetic network, that comprises genes belonging to multiple signalling pathways, notably, Notch, Wnt and FGF (Dequéant et al., 2006; Krol et al., 2011) (Figure 1A). These include Wnt-dependent *Axin2*, FGF signalling pathway genes *Dusp1/2/4/6*, *Snail1/2, Spry2/4*, and Notch pathway genes of the Already defined earlier. HES family, *Lfng* and Nrarp, among others. Strikingly, only two genes–the *Hes1* and *Hes5* orthologs–were conserved in mouse, chicken and zebrafish. Otherwise, the identity of the pathway-specific oscillating genes varied considerably, evidencing evolutionary plasticity of the conserved oscillations in signalling pathway activity (Krol et al., 2011). Several studies have shown that these intercellular communication pathways cooperate during embryo body segmentation. Niwa et al. (2007), (2011) showed that the onset of *Hes7* expression in the mouse tailbud is FGF-dependent, while its maintenance and propagation throughout the PSM requires Notch signalling. A gradient of nuclear Wnt-related β-catenin was shown to control key features of PSM maturation and somite formation (Aulehla et al., 2008). Notch- and Wnt-dependent gene expression oscillations are coupled in the PSM and undergo a phase shift towards the anterior PSM. Inhibition of this phase shift in an *in vitro* setting delayed the arrest of EC waves and impaired tissue segmentation (Sonnen et al., 2018). Identification of EC oscillatory dynamics at the protein level has lagged behind, mostly due to the lack of appropriate antibodies and because it is technically more challenging than the well-established *in situ* hybridization protocols for RNA detection. However, corresponding cycles of protein expression with the same periodicity have been reported and are summarized in Table 3.

**TABLE 2:** Periodicity of gene expression oscillations.

Organism	Gene	Tissue/cell line	Period	Technique	References
Human	*HES1*	UCB1 Mesenchymal stem cells	5 h	qPCR/Microarray	William et al. (2007)
	*HES7*	iPSC	5 h	Live imaging	Diaz-Cuadros et al. (2020)
		PSM-like cells derived from iPSC	∼5 h	Luciferase reporter assay	Matsuda et al. (2020b)
			5.37 h	Luciferase reporter assay	Matsuda et al. (2020a)
		ESC	∼5 h	Luciferase reporter assay	Chu et al. (2019)
Mouse	Axin2	PSM	2 h	*In situ* hybridization (ISH)	Aulehla et al. (2003)
	Dact1	PSM	2 h	ISH	Suriben et al. (2006)
	*Dll1*	PSM	2 h	ISH	Bone et al., 2014; Maruhashi et al., 2005
		NPC	2 h	Live imaging	Shimojo et al. (2008)
	*Dusp4*	PSM	2 h	ISH	Niwa et al. (2007)
	*Hes1*	Myoblasts, fibroblasts, neuroblastoma and teratocarcinoma cells	2 h	qPCR	Hirata et al. (2002)
		C2C12 myoblasts	2 h	qPCR/Microarray	William et al. (2007)
		Fibroblasts (C3H 10T1/2)	2.03 h	Bioluminescence imaging	Masamizu et al. (2006)
		PSM	2.67 h		
		Dissociated PSM cells	2.58 h		
		NPC	2—3 h	Live imaging	Shimojo et al. (2008)
		ESC (MG1.19 cell line)	3—5 h	Live imaging	Kobayashi et al. (2009)
	*Hes5*	Spinal cord cells	3.3 h	Live imaging	Manning et al. (2019)
	*Hes7*	PSM	2 h	ISH	Bessho et al. (2001)
		Induced PSM from ESC	2.5–3 h	Live imaging	Matsumiya et al. (2018)
		PSM-derived cells form iPSC	2.03 h	Luciferase reporter assay	Matsuda et al. (2020a)
	*Lfng*	PSM	2 h	ISH	Aulehla and Johnson, 1999
					Forsberg et al. (1998)
	*Nkd1*	PSM	2 h	ISH	Ishikawa et al. (2004)
	*Notch1*	PSM	2 h	ISH	Bone et al. (2014)
	*Nrarp*	PSM	2 h	ISH	Sewell et al. (2009)
	*Smad6*	Fibroblasts (C3H 10T1/2)	2 h	qPCR	Yoshiura et al. (2007)
	*Snail1*	PSM	2 h	ISH	Dale et al. (2006)
	*Sprouty4*	PSM	2 h	ISH	Hayashi et al. (2009)
	*^(a)^*	PSM	*^(a)^*	qPCR/microarray	Dequéant et al. (2006)
	*^(a)^*	PSM	*^(a)^*	qPCR/microarray	Krol et al. (2011)
Chicken	*hairy2*	PSM	1.5 h	ISH	Jouve et al. (2000)
		Limb bud	6 h		Pascoal et al. (2007)
	*HES4*	limb bud micromass cells	6 h	qPCR	Bhat et al. (2019)
	*HES4*	PSM	1.5 h	ISH	Palmeirim et al. (1997)
	*snail2*	PSM	1.5 h	ISH	Dale et al. (2006)
	*HEY2*	PSM	1.5 h	ISH	Leimeister et al. (2000)
	*LFNG*	PSM	1.5 h	ISH	McGrew et al. (1998)
	*NRARP*	PSM	1.5 h	ISH	Wright et al. (2009)
	*^(a)^*	PSM	*^(a)^*	qPCR/microarray	Krol et al. (2011)
Medaka	*her1/11*	PSM	1 h	ISH	Gajewski et al. (2006)
	*her5*	PSM	1 h	ISH	
	*her7*	PSM	1 h	ISH	Elmasri et al. (2004)
*Xenopus*	*hes5.5*	PSM	0.67 h	ISH	Li et al. (2003)
	*hes9.1*	PSM	0.67 h	ISH	
Zebrafish	*DeltaC*	PSM	0.5 h	ISH	Jiang et al. (2000)
	*her1*	PSM	0.5 h	ISH	Holley et al. (2000)
	*her7*	PSM	0.5 h	ISH	Oates and Ho, (2002)
	*her11*	PSM	0.5 h	ISH	Sieger et al. (2004)
	*her12*	PSM	0.5 h	ISH	Gajewski et al. (2006)
					Shankaran et al. (2007)
	*her15*	PSM	0.5 h	ISH	Shankaran et al. (2007)
	*hey1*	PSM	0.5 h	ISH	Winkler et al. (2003)
	*nrarp-a*	PSM	0.5 h	ISH	Wright et al. (2009)
	*^(a)^*	PSM	*^(a)^*	qPCR/microarray	Krol et al. (2011)

a High throughput study (please refer to original paper for complete gene list); iPSC, induced Pluripotent Stem Cells; ESC, embryonic stem cells; ISH, *in situ* hybridization; PSM, presomitic mesoderm; NPC, neural progenitor cells.

**TABLE 3:** Periodicity of cyclic protein expression.

Organism	Protein	Tissue/cell line	Period	Technique	References
Mouse	Delta1	PSM	2 h	Immunohistochemistry	Bone et al. (2014)
			2.45 h	Live imaging	Shimojo et al. (2016)
		NPC	2.38 h	Live imaging	
		Pancreas	∼1.5 h	Live imaging	Seymour et al. (2020)
	Dusp4	PSM	2 h	Immunohistochemistry	Niwa et al. (2007), (2011)
	Hes1	Myoblasts	2 h	Western Blot	Hirata et al. (2002)
		NPC	2.5 h	Live imaging	Imayoshi et al. (2013)
		Pancreas	∼1.5 h	Live imaging	Seymour et al. (2020)
	Hes5	NPC	2.5 h	Live imaging	Imayoshi et al. (2013)
	Hes7	PSM	2 h	Immunohistochemistry	Bessho et al. (2003)
	NICD	PSM	2 h	Immunohistochemistry	Huppert et al. (2005)
					Niwa et al. (2011)
	Notch1	PSM	2 h	Immunohistochemistry	Bone et al. (2014)
					Morimoto et al. (2005)
	p-ERK	Fibroblasts (CH3 10T1/2)	2 h	Western Blot	Nakayama et al. (2008)
		PSM		Immunohistochemistry	Niwa et al. (2011)
	p-Smad1/5/8	Fibroblasts (CH3 10T1/2)	2 h	Western Blot	Yoshiura et al. (2007)
	Ascl	NPC	2.92 h	Live imaging	Imayoshi et al. (2013)
	Olig2	NPC	6.26 h	Live Imaging	
	Smad6	Fibroblasts (CH3 10T1/2)	2.5 h	Western Blot	Yoshiura et al. (2007)
Chicken	LFNG	PSM	1.5 h	Western Blot	Dale et al. (2003)
Zebrafish	DeltaC	PSM	0.5 h	Immunohistochemistry	Giudicelli et al. (2007)
	Her6	Neural progenitors	1.2–1.4 h	Live imaging	Soto et al. (2020)
	Hes6	PSM	0.5 h	Immunohistochemistry	Schröter et al. (2012)
	Tbx6	PSM	0.5 h	Immunohistochemistry	Wanglar et al. (2014)

PSM, presomitic mesoderm; NPC, neural progenitor cells.

### 3.2 Negative feedback regulation

Several studies have identified negative feedback regulation as a fundamental feature of EC oscillations (Hirata et al., 2002; Bessho et al., 2003; Lewis, 2003; Chen et al.*,* 2005). Figure 1B presents a simplified view of the negative feedback regulatory mechanisms underlying HES gene expression oscillations, whereby *hes* oscillations are maintained by an inhibitory action of the HES protein on its own promoter. HES also inhibits *delta* expression and/or expression of the Notch-modulator Lunatic fringe–*Lfng*, which contributes to synchronized oscillations of Notch-dependent gene expression in neighbour cells. Rapid degradation of the molecular products produced, mRNAs and proteins, ensures propagation of the oscillatory behaviour (reviewed in Kageyama et al., 2012).

Using mathematical modelling, Lewis (2003), Monk (2003) and Jensen et al. (2003) independently postulated that oscillations in gene expression are influenced by delays in the various steps of the regulatory negative feedback loop. Indeed, further experimental evidence showed that the EC mechanism depends on Delayed Negative Feedback loops and that the temporal delays are introduced in multiple steps of the process. Namely:

#### 3.2.1 Transcriptional delay

Lewis (2003) proposed that the time it takes to synthesize a transcript was one of the major accountants for transcriptional delay, so one would assume lengthier genes would have larger transcriptional delays. Elongation was, however, not found to have a major contribution to these delays–RNA polymerase II elongation rate measured in intact zebrafish embryos showed that the time needed to transcribe *her1* and *her7* is negligible and elongation kinetics of *Hes7* and *Lfng* determined using mouse cells also occurred at a fast rate (Hanisch et al., 2013; Hoyle and Ish-Horowicz, 2013). Besides the elongation rate, however, there are other factors that significantly influence the transcriptional delay, namely mRNA nuclear export and mRNA splicing. Giudicelli et al. (2007) experimentally observed a delay from nuclear mRNA production to mature mRNA detection in the cytoplasm of *her1*, *her7* and *deltaC*–zebrafish’s key clock components. Takashima et al. (2011) addressed the contribution of mRNA splicing to gene expression oscillations. They used transgenic mice carrying all or none of *Hes7* introns, together with a luciferase reporter, and assessed the time of *Hes7* transcription and protein production in both conditions. Mice carrying all *Hes7* introns showed a delay of approximately 19 min in *Hes7* expression, when compared to the intron-null mice. When abolishing this delay in a mathematical model, *Hes7* oscillations were abolished, and this was confirmed in the mutant animals. Hoyle and Ish-Horowicz (2013) corroborated that mRNA splicing and nuclear export account for most of the EC transcriptional delay. Additionally, they compared the splicing and export delays in mouse, chicken and zebrafish, and concluded that organisms that have longer delays in these processes also present longer clock periods.

#### 3.2.2 mRNA degradation delay

Another aspect to take into consideration are the half-life times of EC mRNAs and proteins. Due to the inhibitory action of EC products on their own transcription, the time required for their clearance from the cell will directly impact the rate at which a new cycle of gene expression is initiated. Multiple factors that contribute to differential mRNA stability were experimentally assessed for their involvement in EC regulation. These include the mRNA 3′ untranslated region (3′UTR), polyA tail length and microRNA-mediated degradation.

While studying the mechanisms that control segmental gene expression in *Xenopus*, Davis et al. (2001) found that *hes4* (formerly known as *hairy2a*) expression dynamics was influenced by its 3′UTR sequence. When the 3′UTR of *hes4* was substituted by the 3′UTR of other *hes* genes (either from *Xenopus* or other vertebrate species), *hes4* expression retained its characteristic striped pattern in the PSM, unlike what happened when the 3′UTR of constitutively expressed genes was used. The authors further identified a phylogenetically conserved 25 bp sequence in the 3′UTR of EC genes which was necessary and sufficient to confer instability to these transcripts (Davis et al., 2001). Similar findings were reported by Hilgers and colleagues (2005) using an *in vivo* inducible system to halt transcription and measure mRNA degradation rate in the chicken embryo. They clearly showed that the 3′UTR of the EC gene *Lfng* promoted rapid mRNA decay, while the 3′UTR of *fgf8* mRNA contributed to stabilization of the reporter mRNA (Hilgers et al., 2005), which is compatible with *fgf8* graded expression pattern in the PSM (Dubrulle and Pourquié, 2004). Similar findings were reported for zebrafish EC genes (Fujino et al., 2018), evidencing that 3′UTR-mediated regulation of EC gene expression oscillations is a conserved feature in vertebrates. The Amacher lab went on to specify that mRNA decay of both zebrafish *her1* and *deltaC* relies on the Pumilio response- and AU-rich-elements present in their distal 3′UTRs, in a Pnrc2-dependent manner (Gallagher et al., 2017; Tietz et al., 2020).

Different EC genes with the same periodicity in the PSM can nevertheless present very different expression patterns. Nitanda *et al.* (2014) explored this feature focussing on *Hes7* and *Lfng* in the mouse PSM. After bisecting the PSM and culturing one half in actinomycin D to inhibit transcription, while the other was immediately fixed, quantitative PCR analysis showed that *Lfng* mRNA is less stable than *Hes7* mRNA. This was attributed to the 3′UTR, as demonstrated using cells transfected with a reporter vector containing either *Hes7* or *Lfng* 3′UTRs and monitoring mRNA degradation. The authors then generated transgenic mice lines, both containing a reporter gene driven by the *Hes7* promoter, but with different 3′UTRs–one from *Hes7,* and another from *Lfng*. The transgenic line with the *Lfng* 3′UTR showed a severe reduction in reporter mRNA, further confirming the role of the 3′UTR in promoting rapid mRNA decay. Importantly, the reporter mRNA presented the same expression pattern as its 3′UTR-donor gene, i.e., the *Lfng* 3′UTR-reporter displayed the same pattern as endogenous *Lfng*, and this was also true for the *Hes7* 3′UTR-transgene (Nitanda et al., 2014). These results strongly suggest that 3′UTR-mediated mRNA stability defines both the temporal and spatial properties of EC oscillations in the PSM.

Fujimuro et al. (2014) showed that *Hes7* 3′UTR is also required for the production of proper amounts of Hes7 protein to maintain oscillations. In the absence of the 3′UTR, *Hes7* mRNA no longer displayed cyclic expression patterns. The authors found that transcription levels of *Hes7* mRNA were reduced, and that Hes7 protein was hardly detectable in the mouse PSM, compared to wild-type embryos. As expected, since the protein was not being correctly produced, *Hes7* transcription inhibition was impaired, which compromised the maintenance of the oscillations (Fujimuro et al., 2014).

Work performed by Fujino and colleagues (2018) suggested that poly(A) tail length could also be important for EC mRNA rapid turnover. These authors measured the lengths of the poly(A) tails of zebrafish *her1*, *her7* and *hes6*, and observed that the first two genes, that display cyclic expression in the PSM, have shorter poly(A) tails, while *hes6* that is expressed in a gradient has a longer one. Through the inhibition of the deadenylase complex CCR4-NOT, the authors were able to lengthen the poly(A) tails of *her1* and *her7,* and this resulted in a 2-3-fold increase in mRNA levels, indicating an increase in mRNA stability (Fujino et al., 2018).

Finally, EC mRNA degradation rate is also regulated by microRNAs (miRNAs). Xie et al. (2007) were the first to theoretically propose a role for miRNAs in EC delayed negative feedback regulation. Experimental evidence for oscillatory gene modulation by miRNAs was provided by Bonev et al. (2012), who reported that mouse *Hes1* mRNA is a direct target of microRNA-9 (miR-9). *Hes1* oscillations were dampened either when mir-9 was overexpressed or its binding to *Hes1* was inhibited, suggesting that *Hes1* oscillations are maintained within a certain range of miR-9 levels. This is ensured by negative feedback of Hes1 on the production of miR-9 primary transcripts, generating a double-negative feedback loop. Although the pri-miR-9 and pre-miR-9 are processed and cleared at a fast rate, the same is not true for the mature miR-9 which accumulates in the cell over time. Hence, a self-limiting oscillator model was proposed, whereby when miR-9 levels reach a certain threshold, *Hes1* is permanently downregulated and NPC differentiation occurs (Bonev et al., 2012). Similar findings were further reported in zebrafish hindbrain development. Here, miR-9 acts on *her6* to ensure robust oscillatory expression during neural progenitor cell differentiation (Soto et al., 2020).

During somitogenesis, miR-125a-5p is expressed in the chicken PSM where it targets the *Lfng* 3′UTR (Riley et al., 2013). Inhibition of chicken miR-125a-5p activity resulted in abnormal somite segmentation, resembling the phenotype obtained when *Lfng* was ubiquitously expressed in the chicken PSM (Dale et al., 2003). This is consistent with a role for miR-125a-5p in promoting *Lfng* mRNA decay. Moreover, *Lfng* and *hairy1* lost their oscillatory expression pattern, further evidencing that miRNA-mediated regulation is necessary for EC gene expression oscillations (Riley et al., 2013). A regulatory action of miR-125a-5p on *Lfng* mRNA degradation and expression dynamics was also documented in the mouse embryo (Wahi et al., 2017). Mathematical modelling performed by Jing et al. (2015) provided important insights regarding miRNA role in the segmentation clock. Their work suggests that the interaction between *Lfng* and miR-125a-5p affects both the amplitude and period of the oscillations, thus acting as a fine-tuning mechanism to Notch activity during somitogenesis.

Despite the established importance of miRNAs for mRNA decay, the extent of their relevance for EC oscillations is still unclear. Recent work from our group showed that different miRNA species are expressed in the PSM and in the forelimb distal cyclic domain. These tissues have very different EC periodicities (discussed below), which suggests that miRNAs may play a role in establishing different paces of the EC (Duarte et al., 2022).

#### 3.2.3 Protein turnover delay

Even though translational delays are not accounted to influence oscillations (Hoyle and Ish-Horowicz, 2013), protein stability plays a crucial role. Hirata et al. (2004) addressed what would happen if Hes7 protein half-life time increased from 20 min (wild-type conditions) to 30 min and found that this provokes a dampening in both Hes7 mRNA and protein oscillations over time. Interestingly, lysine residues were found to play a key role in Hes7 protein stability. The authors generated Hes7 protein mutants, by introducing lysine-to-arginine mutations for each of the seven lysine residues in Hes7 sequence and found that different mutations gave rise to proteins with a half-life that differed from the wild-type. Ishii et al. (2008) reported that some of the lysine mutants lost transcriptional repressor activity, although they were more stable than the wild-type counterpart, thus evidencing the role of these lysine residues in Hes7 protein stability. Studies done by Lewis (2003) and Giudicelli et al. (2007) also stated that *her* protein half-life time should be short, compared to the zebrafish’s segmentation clock pace. Mathematical modelling performed by Ay et al. (2013) reiterated the finding that proteins with a short half-life time are an essential requirement for the maintenance of the period of oscillations in the wild-type zebrafish segmentation clock. They further confirmed this by determining that Her7 protein has a half-life time ∼10 times inferior to the zebrafish segmentation clock period (Ay et al., 2013).

### 3.3 Cell autonomous *vs*. tissue level oscillations

Embryo Clock gene expression oscillations are a cell autonomous property. This was first hinted by dissecting the chicken PSM in multiple portions and observing that the overall expression pattern of *hes4* (Palmeirim et al., 1997) and *Lfng* (Maroto et al., 2005) remained intact. The same was observed in dissected mouse PSMs (Masamizu et al., 2006). Cyclic *Lfng* gene expression even persisted in dissociated chicken PSM cells, but it occurred asynchronously among cells, evidencing the need for cell-cell contact to ensure synchrony and establish robust cyclic expression patterns at the tissue level (Maroto et al., 2005). In dissociated mouse PSM cells, *Hes1* oscillations also occur cell-autonomously (Masamizu et al., 2006) and Webb et al. (2016) further reported that zebrafish *her1* gene retains oscillatory expression in isolated tailbud cells. In this case, oscillations in individual cells presented a longer period and were less robust, compared with the intact tissue. Altogether, these results suggest that cell-cell communication is a key requirement for oscillations to be in phase within the vertebrate PSM tissue. This was corroborated by the work of Tsiairis and Aulehla (2016), that showed that a re-aggregation of mouse PSM cells is able to synchronize oscillations. Cell-autonomous EC gene expression oscillations have also been described in other cell types, such as mouse embryonic stem cells (ESC) (Kobayashi et al., 2009), individual fibroblasts (Masamizu et al., 2006) and neural progenitor cells (Shimojo et al.*,* 2008; Bonev et al*.,* 2012; Manning et al., 2019). These can be synchronized *in vitro* by the application of a serum shock or by Notch activation (Hirata et al., 2002). However, cell-specific distinct phases of EC oscillations may also play important roles *in vivo*. This will be discussed further below.

EC synchronization between PSM cells is required for proper somite formation. Local synchrony within the PSM tissue is achieved through Delta-Notch signalling (Jiang et al., 2000; Horikawa et al*.,* 2006; Riedel-Kruse et al., 2007), which also functions to overcome the effect of “noise” introduced by other biological processes, such as cell division (Horikawa et al., 2006; Riedel-Kruse et al., 2007). Riedel-Kruse and colleagues found that EC synchrony in the zebrafish embryo is achieved by simultaneous (Notch-independent) activation immediately prior to gastrulation and is then maintained by Notch-dependent self-organized synchronization. The latter was elegantly shown by incubating embryos with the Notch-inhibitor DAPT until complete EC desynchronization. Then, DAPT washout alone was sufficient to completely restore both *dlc* oscillations and somite formation (Riedel-Kruse et al., 2007). Delaune et al. (2012) applied single cell live-imaging of *her1* expression in zebrafish wild-type and mutant embryos for *deltaC*, *deltaD* and *notch1a* to study the role of Delta-Notch signalling in EC synchronization. In the mutants, *her1* dynamics persisted in PSM neighbour cells, but in different oscillation phases (Delaune et al., 2012). Interestingly, *deltaC* and *deltaD* work together to ensure synchrony of the zebrafish segmentation clock in distinct portions of the PSM. While *deltaD* is responsible for the onset of the oscillations at the tailbud level, *deltaC* plays in role in maintaining and amplifying the oscillations in adjacent cells along the PSM tissue (Mara et al., 2007).

Soza-Ried et al. (2014) provided conclusive evidence for the role of oscillations of Notch-Delta signalling in maintaining the EC synchronized in neighbour cells for somite segmentation. Using a *deltaC* zebrafish mutant line, the authors were able to rescue both *her1* oscillations and somite formation by applying short artificial pulses of *deltaC* expression, evidencing that Notch signalling is indeed maintaining cell synchrony during somitogenesis. Accordingly, longer intervals between *deltaC* pulses generated larger somites (Soza-Ried et al., 2014). This was confirmed by Isomura et al. (2017) who developed an optogenetics-based system to monitor Notch-Delta signalling dynamics in neighbour cells. Light-induced *Dll1* pulses in sender cells were able to generate synchronized oscillations of *Hes1* expression in receiver cells. Furthermore, they were able to determine the time from the induction of *Dll1* to the cleavage of NICD, which was ∼50.9 min, followed by an additional ∼77 min until maximum *Hes1* levels were reached (Isomura et al., 2017). More recently, Yoshioka-Kobayashi et al. (2020) used this system to show that LFNG in sender cells introduces a 15 min-delay in the transport of Dll1 protein to the cell membrane, without which HES7 oscillations are severely dampened in individual cells of the PSM. These studies corroborate the importance of delays in cell-cell communication for EC oscillations and illustrate the power of optogenetics-based tools for dissecting these intricate regulatory mechanisms.

Notch-Delta signalling was shown to require non-muscle myosin II (NM II)-dependent contractility in both signal-sending and -receiving cells (Hunter et al., 2019). Recently, our lab evaluated the importance of fibronectin (FN) extracellular matrix assembly and signalling through the integrin-ROCK-NM II axis for somite segmentation and EC oscillations in chick PSM. We found that experimental treatments targeting FN matrix assembly, cell-FN interactions and actomyosin contractility significantly perturbed somite formation and EC gene expression, highlighting the importance of the PSM tissue’s mechanical properties for EC oscillations (Gomes de Almeida et al., 2022).

*Hes7* and *her1* oscillations slowdown in the anterior PSM in mouse (about 1.5-fold) (Niwa et al., 2011) and in zebrafish (Giudicelli et al., 2007; Delaune et al., 2012). Shih et al. (2015) corroborated these findings using live imaging in a transgenic zebrafish line with a her1-venus reporter. They saw that the periodicity of the segmentation clock increases by 1.5-fold in the anterior PSM, comparatively to the posterior PSM. SSoroldoni et al. (2014) had previously described this as a Dynamic Wavelength effect that, together with a Doppler effect resulting from the relative motion of the anterior PSM towards the posterior end due to tissue shortening over time, explains the rhythm of embryo body segmentation. To better understand the dynamics of EC deacceleration in the anterior PSM, Shih et al. (2015) assessed her1-venus reporter expression in cells that would form either side of a somite boundary. Within the same presumptive somite, clock oscillations were arrested in a posterior-to-anterior direction, i.e., cells that were incorporated in a posterior somite boundary ceased oscillations prior to cells that were incorporated in the anterior boundary of the same somite. Moreover, the authors reported that cells at a one-somite distance are initially synchronized in the posterior PSM and, as the clock slows down in the anterior PSM, they assume opposite phases of EC expression (Shih et al., 2015).

Another important alteration that cells experience as they transition from the posterior to anterior PSM is the relative timing of Notch- and Wnt-dependent EC oscillators (Sonnen et al., 2018). In fact, *Lfng* (Notch) and *Axin2* (Wnt) oscillated out-of-phase in the posterior PSM and were progressively coupled towards the anterior PSM, where their synchronization was critical for somite segmentation. This was shown using an ingenious microfluidics-based system, which allowed for precise manipulation of gene expression oscillations by applying temporally controlled pulses of Wnt/Notch-specific activator molecules (Sonnen et al., 2018). Recently, it was shown that the distinct levels of FGF signalling experienced in the anterior and posterior regions of the PSM could underlie the differences in EC dynamics observed in these cells (Diaz Cuadros et al., 2020; Yaman et al., 2022). Yaman et al. (2022) further reported that the posterior-to-anterior FGF gradient in the PSM, classically solely associated with the *wavefront* activity, is also controlling the anterior propagation of the EC oscillations (Yaman et al., 2022).

There is also evidence of significant spatiotemporal metabolic changes along the PSM axis. Microarray analysis performed in PSM and tailbud samples of zebrafish embryos revealed that cell cycle/DNA metabolic functions are enriched in the posterior PSM, while translation/oxidative metabolism is enriched in anterior PSM and somites. The authors also reported a 2-fold increase in ATP content, as well as 2.5-fold decrease of Cytochrome C oxidase activity in the posterior PSM compared to anterior tissues (Özbudak et al., 2010). A posterior-to-anterior gradient of glycolytic activity was also linked to presomitic mesoderm development in mouse (Bulusu et al., 2017) and chicken (Oginuma et al., 2017) embryos. To test the functional relevance of these metabolic differences, PSM explants were cultured in glucose- or pyruvate-supplemented medium. While explants cultured in glucose supplemented medium developed normally, pyruvate-cultured explants displayed several defects concomitantly with loss of *Lfng* gene expression in the posterior PSM. Using a genetically encoded sensor for pyruvate to monitor metabolic transitions during PSM differentiation in real-time, the authors reported that pyruvate levels, i.e., glycolytic activity, decreased as cells transited towards an anterior PSM-like, more differentiated, state. Consistent with these findings, chicken embryos treated with 2-deoxy-D-glucose (2DG), a competitive inhibitor of the glycolytic enzyme hexokinase, displayed severe elongation defects, even though somite formation occurred normally. On the other hand, embryos treated with sodium azide (NaN_3_)—a respiration inhibitor, had impaired somite segmentation (Oginuma et al., 2017).

## 4 Embryo clock periodicity

### 4.1 Different species

**FIGURE 2:**
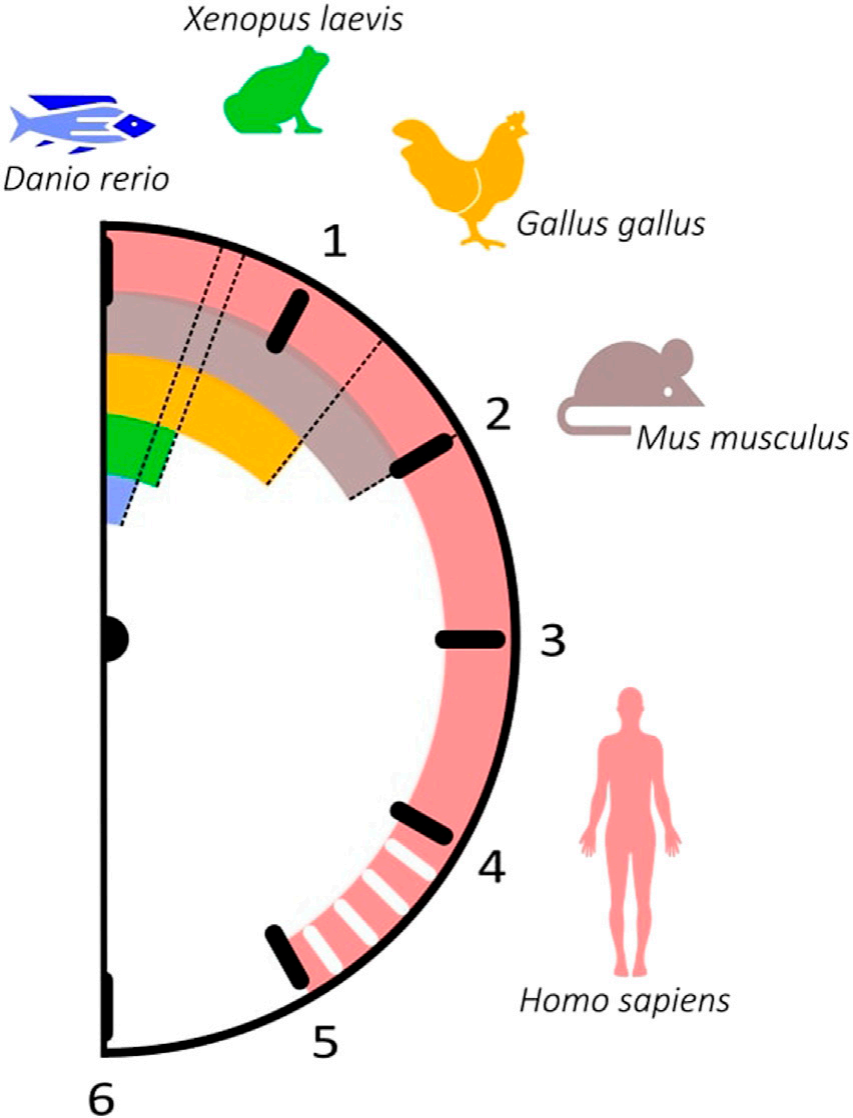
The somitogenesis clock ticks with different paces among vertebrates. The periodicity of somitogenesis clock gene expression in different organisms correlates with somite formation time. *Danio rerio*: 30 min (blue); *Xenopus laevis:* 40 min (green); *Gallus gallus*: 90 min (orange); *Mus musculus*: 120 min (brown); *Homo sapiens*: ∼5 h (pink).

The time each pair of somites takes to form is species-specific and displays great variability between organisms, ranging from 30 min in zebrafish to approximately 5 h in Human (Table 1). Similarly, the expression of segmentation clock genes oscillates with a periodicity characteristic of each species, which closely matches the time of somite formation (Figure 2; Table 2). The signalling pathways that comprise these genes are conserved; however, data suggests that cyclic genes display an evolutionary plasticity, since the specific genes involved in each pathway differ in the studied organisms (Krol et al., 2011).

A curious aspect of somite formation time is that the size of the organism does not significantly influence the time a pair of somites takes to form. For instance, somites have a relatively similar time of formation in the chicken and the emu, although dimension-wise these two birds are very distinct (Nagai et al., 2011). Likewise, the time of somite formation does not depend on phylogenetic relationships, since vertebrates belonging to different phyla can have the same somitogenesis period: 60 min for medaka and the house snake (Gomez et al., 2008).

Recent work started shedding light into the molecular mechanisms underlying divergent EC periodicity among organisms. Using *in vitro* models to compare EC gene expression dynamics in mouse *vs*. human cells, two independent studies found that the near 2-fold difference in oscillation periodicity could be explained by the different speeds in biochemical reactions within human and mouse cells, in particular mRNA and/or protein decay rates (Matsuda et al., 2020a; Rayon et al., 2020). This was documented in different cell types, which suggests that global temporal scaling mechanisms are a cell-autonomous property of the organism (Rayon and Briscoe, 2021).

### 4.2 Different axial levels of the same organism

**FIGURE 3:**
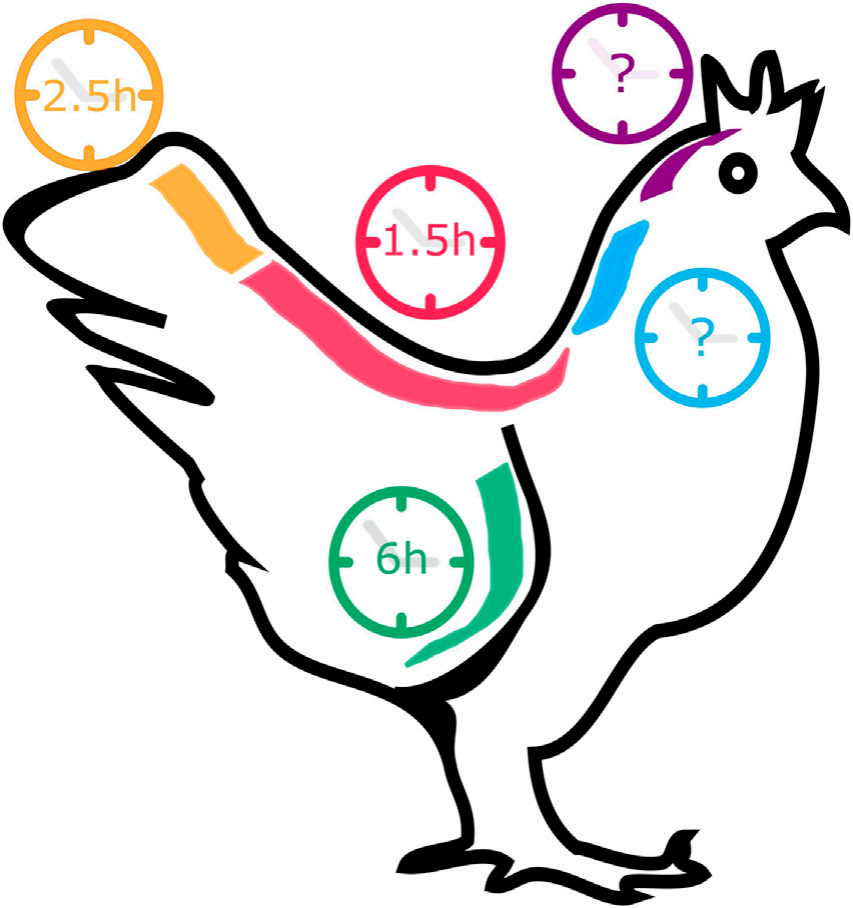
The pace of *Gallus gallus* Embryo Clock (EC) oscillations in different tissues. During somitogenesis (red and orange), the EC pace ranges from 1.5 to 2.5 h, while during forelimb development (green) a cycle lasts 6 h. EC dynamics in the early stages of somitogenesis (blue) and in neural development (purple) remain unknown.

EC oscillations underlying the formation of somites positioned at different A-P levels of the vertebrate body axis have different periodicities. In the chicken embryo, somitogenesis and EC oscillations occur with a 90 min-periodicity for somites 15–20 (HH12-13^+^) (Palmeirim et al., 1997). However, the final 5-8 somites (HH23) form with a periodicity of 150 min, matched by correspondingly slower cycles of *Lfng* gene expression (Gibb et al., 2009; Tenin et al., 2010) (Figure 3). In the opposite end, knowledge on the EC in the formation of the anterior-most somites is scarce. Rodrigues et al. (2006) characterized the expression of Notch-related EC genes in somites 1–10 and reported that, while they were dynamically expressed in the PSM, they did not present somite A-P polarity, as occurs in caudal somites. The EC was proposed to already be active even earlier in development, during gastrulation. In fact, Jouve et al. (2002) reported the existence of pulses of gene expression of *hairy2* and *Lfng* in the prospective PSM of early chicken embryos. In zebrafish gastrulation stages, the EC already oscillates with 30-min periodicity (Riedel-Kruse et al., 2007) and in mouse, with a ∼2-h period (Falk et al., 2022). What triggers the onset of the Embryo Clock, and the existence of a clear periodicity in the early developmental stages of chick development, however, remains elusive.

Differences regarding segmentation clock operation in anterior and posterior regions of the zebrafish body axis have also been described (Choorapoikayil et al., 2012; Schröter et al., 2012; Hanisch et al., 2013). Although the double-mutant and -morphant for *her1* and *her7* exhibit defects throughout the entire body axis (Henry et al., 2002; Oates and Ho, 2002; Lleras-Forero et al., 2018), disrupting *her1* or *her7* gene expression has a different impact depending on the somites that are being formed. In fact, segmentation defects in *her1* mutants are restricted to the anterior trunk, while in *her7* mutants somites become defective only posterior to somite 8 (Choorapoikayil et al., 2012; Schröter et al., 2012; Hanisch et al., 2013).

### 4.3 Different tissues of the same organism

Throughout development, the same gene regulatory networks can be employed in different tissues to produce multiple outcomes. Besides the segmentation of the axial vertebrate body plan, EC oscillations also participate in other developmental processes, where their dynamics differs from the one presented in the PSM during somitogenesis.

Embryonic stem cells (ESC) differentiate into cell types belonging to all three germ layers–mesoderm, endoderm and ectoderm. Evidence that oscillatory gene expression played an important role in ESC differentiation was first provided by Kobayashi et al. (2009). The authors identified unsynchronized cycles of *Hes1* gene expression in mouse ESCs with a periodicity of 3–5 h–longer than the 2-h *Hes1* period described for other cell types from this organism (see Table 2). Under the same culture conditions, Hes1-high cells differentiated more efficiently into mesodermal cells, while Hes1-low cells into neurons, suggesting that unsynchronized gene expression oscillations might potentiate heterogeneous cell fate specification within the same population of cells (Kobayashi et al., 2009).

Oscillatory EC expression was also described in mouse neural progenitor cells (NPC). Using real-time imaging, *Hes5* and *Dll1* mRNA were determined to oscillate with a 2 h-periodicity (Imayoshi et al., 2013; Shimojo et al., 2008, 2016), and for *Hes1* this period was 2–3 h (Shimojo et al., 2008; Imayoshi et al., 2013) (Table 2). Likewise, Hes1 and Delta1 proteins display an identical period of oscillation (Table 3; Imayoshi et al., 2013). Studies performed in mouse NPCs reiterated the importance of gene expression oscillations for cell fate determination. During neurogenesis, the NPC population is maintained due to the repression of neural fate determination factors, such as *Neurogenin2*, *Ascl1/Mash1* and *Olig2* by *Hes1* and *Hes5* oscillatory expression levels. As a consequence, *Neurogenin2* and *Ascl1* also display oscillatory mRNA expression (Shimojo et al., 2008; Imayoshi et al., 2013). Unlike somitogenesis, oscillations during neural development are asynchronous. While undergoing differentiation, neural cells impede their neighbours to differentiate into the same cell type through lateral inhibition mediated by the Notch signalling pathway. Imayoshi et al. (2013) reported that the expression level of gene oscillations plays an important role in NPC differentiation. Upon segregating NPC according to their levels of expression of *Hes1*, *Ascl1* and *Olig2* and culturing them in differentiation medium, the authors found that high or low EC expression levels dictated different differentiation outcomes. For instance, Hes1-high NPCs differentiated into an astrocyte lineage, while Hes1-low NPCs into neurons (Imayoshi et al., 2013).

Similar to what is observed during neural development, *Hes1* expression is required for the maintenance of multipotency in pancreatic progenitors, and undifferentiated neighbour cells undergo a mechanism of lateral inhibition to give rise to different cell types (de Lichtenberg et al., 2018). Even though pancreas development shares common players with somitogenesis and neural development, it was unknown if they displayed an oscillatory behaviour in this tissue. Recently, Seymour and colleagues (2020) reported that *Hes1* and *Dll1* proteins oscillate with a 90-min periodicity in cultured mouse pancreatic explants, and that this stimulates progenitor proliferation. The periodicity is different from the average 150 min period of Hes1 oscillations in NPC, which could be explained by lower levels of Notch activation in pancreatic progenitors. Importantly, extending the Hes1 oscillation period to ∼120 min by inhibiting NICD degradation altered cell fate specification (Seymour et al., 2020).

Oscillatory gene expression was also reported during chicken limb development (Pascoal et al., 2007) (Figure 3). Oscillations of *hairy2* expression were first described in the chicken PSM, with the same periodicity as somite formation–90 min (Jouve et al., 2000). To study *hairy2* expression dynamics in the developing chick forelimb, Pascoal et al. (2007) microsurgically removed one limb from HH22-26 embryos *in ovo* and reincubated the embryo for different periods of time. *hairy2* expression was then assessed in each limb pair using *in situ* hybridization, revealing that *hairy2* has very dynamic expression in the distal limb field, that is recapitulated every 6 h. The authors then determined that the time required to form a new autopod skeletal element is 12 h, suggesting that the limb chondrogenic precursor cells undergo two cycles of *hairy2* expression for the formation of each autopod segmented element (Pascoal et al., 2007). This was the first evidence that a molecular clock is operating during limb development, a process where temporal control is also fundamental. *hes4* is also expressed in the distal mesenchyme of the avian limb (Vasiliauskas et al., 2003) and recent work suggests that its expression is also cyclic during limb development Bhat et al. (2019). cultured cells from chicken pre-cartilage leg mesenchyme and observed oscillations of *hes4* expression with a period of 6 h, suggesting that EC periodicity is a tissue-specific property.

The cases mentioned above clearly exemplify that the EC can play very distinct roles in different cells and tissues. For the formation of segmented structures such as somites and autopod limb elements, cells need to be synchronized to aggregate and give rise to a new segment. In multipotent cells, such as ESC, NPC and pancreatic progenitors, asynchronous EC oscillations function to allow heterogeneous cell fate responses of the population to a differentiation signal, ensuring the simultaneous specification of multiple cell types required for normal development.

## 5 Experimental manipulation of the Embryo Clock

Many attempts have been made to manipulate EC gene expression levels and/or temporal dynamics in order to obtain a clear understanding of the mechanisms underlying ultradian biological rhythms and their impact on embryo development. Although EC periodicity can be significantly altered throughout the developmental program (Figure 3)–chicken *hairy2* oscillates with a periodicity of 90 min in embryos with 48 h (PSM) (Palmeirim et al., 1997) and 6 h in the forelimb of older embryos (4–5 days) (Pascoal et al., 2007)–it has been extremely challenging to produce such significant alterations in an experimental setting. Most of the attempts to date completely disrupted EC expression or oscillatory dynamics (Table 4). In the most cases, only slight alterations to its rhythmicity were obtained (Table 5). The knowledge gained by such approaches, however, has been invaluable, and is patent in the topics described in the previous sections of this review.

**TABLE 4:** Experimental disruption of Embryo Clock dynamics.

Organism	Tissue	Manipulation	Somitogenesis phenotype	Altered gene expression	References
Mouse	Embryo	Hes7 KO	Segmentation and skeletal patterning defects	Hes1, Hey2, Lfng, Sprouty4, Nrarp and Nkd1 disrupted oscillations. Steady expression of NICD and MESP2	Besho et al. (2001), Besho et al. (2003); Hayashi et al. (2009), Ferjentsik et al. (2009), Ishikawa et al. (2004), Niwa et al. (2007), (2011)
	Embryo	Hes7 overexpression	n/a	Sprouty4 absent in the posterior PSM and static expression in the anterior PSM	Hayashi et al. (2009)
	PSM	hes7 ± and Mesp2^+/−^mutants in mild hypoxia	Segmentation and skeletal patterning defects	Notch pathway and FGF are downregulated	Sparrow et al. (2012)
	Embryo	Hes7 intron deletion	Fused somites and skeletal patterning defects	Sustained Hes7 expression	Takashima et al. (2011)
	Embryo	Hes7 3′UTR insertion of 5, 10 or 20 kb	Segmentation and skeletal patterning defects	LFNG and Hes7 dampened oscillations	Fujimuro et al. (2014)
	Embryo	Dll1 KO	Segmentation and skeletal patterning defects	Lfng and Hes7 expression absent	Barrantes et al. (1999), Chen et al. (2005), Niwa et al. (2007), Zhang et al. (2002)
	Embryo	Dll1 gene shortening/elongation	Fused somites	Steady Dll1 protein expression and dampened oscillations of Hes1 and Hes7	Shimojo et al. (2016)
	Embryo	Dll3 KO	Severe segmentation defects	Lfng, Hes1 and Hes5 absent expression. Steady Hes7 and Nrarp expression	Chen et al. (2005), Dunwoodie et al. (2002), Sewell et al. (2009)
	Embryo	RBPJκ KO	n/a	Lfng expression absent	Barrantes et al. (1999)
	Tailbud explants	Uncoupled notch and wnt oscillations	Halted segmentation	Delayed arrest of oscillations	Sonnen et al. (2018)
	Embryo	Lfng KO	Somite defects and axial strutures defects	Hes7, NICD and Nrarp with disrupted oscillatory expression	Chen et al. (2005), Ferjentsik et al. (2009), Morimoto et al. (2005),Niwa et al. (2007), (2011), Sewell et al. (2009), Shifley et al. (2008)
	Embryo	Lfng overexpression	Segmentation and skeletal patterning defects	Steady Hes7 expression	Serth et al. (2003)
	Embryo	LFNG dominant alele (RLFNG) resistant to Golgi degradation and non secreted	Absent or disorganized intersomitic boundaries	Abolished Dll1, Notch and Hes7 oscillations	Williams et al. (2016)
	Embryo	wnt3a vt mutant	Segmentation and skeletal patterning defects	Axin2 and Nrarp expression absent. Lfng and Hes7 oscillations abolished	Aulehla et al. (2003), Nakaya et al. (2005), Niwa et al. (2007), Sewell et al. (2009)
	Embryo	Ctnnb1 KO	Defective somites and boundaries	Axin, Dusp6/Mkp3, Spry2, Lfng and Hes7 with very low or no expression	Dunty et al. (2008)
	Embryo	Fgfr1 cKO (driven by T promoter)	Segmentation and skeletal patterning defects	Hes7 expression absent; Lfng steady expression; Dusp4, Sprouty4, Axin2 and Snail1 are downregulated	Niwa et al., 2007; Wahl et al., 2007
	Embryo	Psen1 KO; Psen2 KO	Do not form any somites	NICD, Snail1 and Sprouty2 with absent expression; Hes7, Axin2 and Dusp6 are expressed only in the tailbud	Ferjentsik et al. (2009)
Chicken	Embryo	Mir-125-5p manipulation (target protection assay)	Absent or disorganized intersomitic boundaries	Steady hairy1 expression and absent Lfng expression	Riley et al. (2013)
	Forelimb	Abrogate FGF signaling via AER ablation or inhibiting drugs	n/a	Absent hairy2 expression in the Distal Cyclic Domain	Sheeba et al. (2012)
	Forelimb	Abrogate Shh signaling via ZPA ablation or inhibiting drugs	n/a	Absent hairy2 expression in the Distal Cyclic Domain	
Medaka	Embryo	Pharmacological modulation of ROS levels (NAC and DPI treatment)	Defective somites and boundaries	her4 and hey1 downregulated	Ventre et al. (2015)
Zebrafish	Embryo	her1 MO	Somite boundary defects	Steady deltaC, her7 and mesp2 expression	Gajewski et al. (2003), Sieger et al. (2004), Shankaran. (2007); Holley et al. (2002)
	Embryo	her7 MO	Somite boundary defects	Steady deltaC, her1, her 11, her12, her 15 and mesp2 expression	Gajewski et al. (2003), Sieger et al. (2004), Shankaran et al. (2007), Trofka et al. (2012)
	Embryo	her1 and her7 double mutant	Defective somite shape	Constant deltaC expression in the anterior PSM	Lleras-Forero et al. (2018)
	Embryo	her1, her7 and hes6 triple mutant	Defective somite shape	Constant deltaC expression in the anterior PSM	
	Embryo	her1, her7 and Tbx6 triple mutant	Defective somite shape	Constant deltaC expression throughout the PSM	
	Embryo	her1 and her7 double MO	Defective somites and boundaries	deltaD, Mesp2 and Notch expression disrupted	Henry et al. (2002), Oates and Ho (2002)
	Embryo	her1 mutant	disrupts the three anterior-most somite borders	Steady deltaC, her1, her7 and mesp2 expression	Choorapoikayil et al. (2012), Schröter et al. (2012), Hanisch et al. (2013)
	Embryo	her7 mutant	somite border defects from somite 8 to 17	Steady deltaC, her1, her7 and mesp2 expression	
	Embryo	her1 and deltaC double mutant	Defective somites and boundaries	Her7 expression through all PSM	Choorapoikayil et al. (2012)
	Embryo	deltaC MO	Defective somites and boundaries	Constant Her1 expression	Holley et al. (2002)
	Embryo	deltaC mutant (*bea*)	Defective somites and boundaries	Constant Her1 expression	Choorapoikayil et al. (2012), Holley et al. (2000),(2002)
	Embryo	deltaD mutant (*aei*)	Defective somites and boundaries	her12 and her15 absent expression. her1 and her11 with static expression	Sieger et al. (2004), Shankaran et al. (2007), Holley et al. (2000)
	Embryo	her12 overexpression	Defective somites and boundaries	Constant her1, her7 and deltaC expression	Shankaran et al. (2007)
	Embryo	her15 overexpression	Defective somites and boundaries	Constant her1, her7 and deltaC expression	
	Embryo	her12 MO	n/a	Constant her1, her7 and deltaC expression	
	Embryo	Notch1 mutant (des)	Defective somites and boundaries	her12 and her15 downregulation. Static her1, her7 and her11 expression	Sieger et al. (2004), Shankaran et al. (2007), Holley et al. (2000)
	Embryo	NICD activation	Somite boundary defects	difuse her1 and her7 expression	Ozbudak and Lewis. (2008)
	Embryo	Su (H) MO	Defective somites and boundaries	her12 and her15 downregulation. Static her1, her7 and her11 expression	Sieger et al. (2003), (2004)
					Shankaran et al. (2007)
	Embryo	Greb1 MO	Defective somites and boundaries	Downregulated her7	Prajapati et al. (2020)
	Embryo/hindbrain	Mutation of the miR-9 target site on her6 3′UTR	n/a	Stabilized her6 levels	Soto et al. (2020)
	Embryo	her1/her7 disrupted chromossomal linkage	Defective somites and boundaries	Constant her1 and her7 expression	Zinani et al. (2021)

**TABLE 5:** Embryo Clock pace manipulation.

Organism	Manipulation	wt pace	Altered pace	Δ pace	References
Mouse	Deletion of Hes7 introns 1 and 2	123 min	112 min	(-) 8,94%	Harima et al. (2013)
	Hes1 type-1 mutant (NPC)	173.5 ± 4.4 min	159.9 ± 2.6 min	(-) 7,8%	Ochi et al. (2020)
	Hes1 type-2 mutant (NPC)		187.0 ± 4.3 min	(+) 7,8%	
	Hes7 K14R mutation (HES7 prot half-life increase from 20 to 30 min)	121.4 min	131.6 min	(+) 8,4%	Hirata et al. (2004)
	KO of Nrarp	106 min	111 min	(+) 4,5%	Kim el al. (2011)
	LiCl 20 mM treatment	2.5 h	2.9 h	(+) 16%	González et al. (2013)
	LiCl 40 mM treatment	2.5 h	3.6 h	(+) 44%	
	CKI-7 100 µM treatment	2.5 h	3.3 h	(+) 32%	
	pancreatic dorsal bud, MLN4924 treatment (NICD stabilization)	∼90 min	∼120 min	(+) 33%	Seymore et al. (2020)
	PSM-like tissue (iPSM)	159.6 min	^a^ 123.3–203.3 min	^a^ (-) 22.7% - (+) 25.7%	Yoshioka-Kobayashi et al. (2020)
Zebrafish	Damascus mutant (∼100 deltaD copies)	24.7 ± 0.6 min	23.1 ± 0.8 min	(-) 6.4%	Liao et al. (2016)
	MO hes6	n/a	n/a	(-) 6.5% ± 1.2%	Schroter and Oates. (2010)
	Mib1 mutant	n/a	n/a	(+) 19%	Herrgen et al. (2010)
	aei/deltaD mutant	n/a	n/a	(+) 23%	
	des/notch1a mutant	n/a	n/a	(+) 7%	
	Notch inhibition with saturating DAPT concentrations (R 40 mM)	n/a	n/a	(+) 18%	
	Her7 hetero:hes6 mutant	n/a	n/a	(+) 6%	Schröter et al. (2012)
	Her7 Mutant:hes6 mutant	n/a	n/a	(+) 5%	
Chicken	CKI-7 100 µM treatment	90 min	115–120 min	(+) 33%	Gibb et al. (2009)
	Shh inhibition/notochord removal	90 min	∼2 h 45 min	(+) 85%	Resende et al. (2010)
	Blebbistatin 50 µM treatment	90 min	120 min	(+) 33%	Gomes de Almeida et al. (2022)

n/a: data not available.

a Chemical library screening; maximum range is indicated (please refer to original paper for complete list and respective alterations).

**FIGURE 4:**
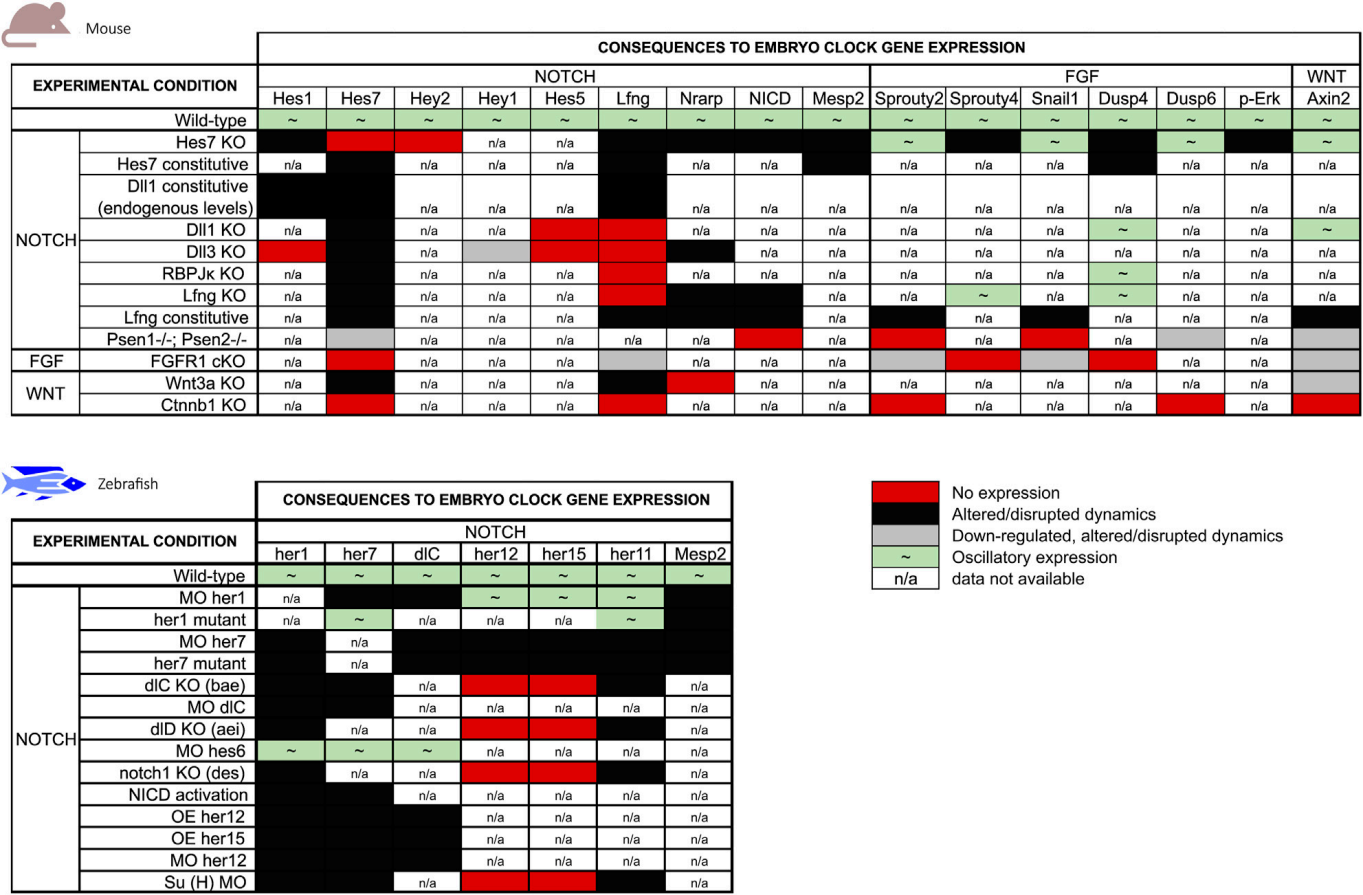
Summary of the effects of genetic manipulation on mouse and zebrafish EC gene expression. EC genes were grouped by the main signalling pathways they are associated with.

Genetic manipulation of EC genes and associated intercellular signalling pathways provided the main framework of what we know today. Figure 4 offers a graphical overview of the alterations to EC gene expression imposed by genetic manipulation in the mouse and zebrafish models (references listed in Tables 4, 5). An interesting observation is that manipulation of Notch-dependent EC genes has limited impact on the dynamics of oscillatory genes associate with the FGF or Wnt signalling pathways, while the other Notch-EC genes are significantly altered. The major effects on FGF clock genes were observed when *Hes7* or *Lfng* were expressed at constant levels and only the latter altered Wnt-related *Axin2* oscillations. On the contrary, modulation of key components of FGF and Wnt pathways significantly impacted the expression of EC genes pertaining to all signalling pathways (Figure 4). The available information in zebrafish regards only to Notch-pathway EC genes and provides complementary knowledge to what is described for mouse. As can be easily perceived from Figure 4, many more studies are required to make full sense of the information gathered to date and to allow a clear inter-species comparison of the EC mechanism. It is worth highlighting that conclusive evidence for the functional relevance of the dynamic nature of EC gene expression, in opposition to EC expression levels, was provided by Shimojo et al. (2016). These authors succeeded in abolishing *Dll1* oscillations while ensuring physiological expression levels of the protein and this led to defective somitogenesis.

Besides genetic manipulation, other factors, such as environmental hypoxia or alterations to reactive oxygen species (ROS) levels, can impact EC operation in the PSM. Exposure of pregnant mice to mild hypoxia disrupted EC oscillations and induced vertebral malformations in heterozygous pups for *Hes7* or *Mesp2*, which otherwise developed normally (Sparrow et al., 2012). Ventre et al. (2015) suggested that this effect could be mediated by ROS, since pharmacological modulation of ROS levels in Medaka (*Oryzias latipes*) impaired somite formation and downregulated *her4* and *hey1,* two EC genes in this organism (Ventre et al., 2015). Recent studies using *in vitro*-derived PSM-like tissues showed that graded levels of Fgf ligands are required to ensure the EC dynamics (pace, amplitude and phase) (Diaz Cuadros et al., 2020; Yaman et al., 2022) and directionality of EC oscillation waves in the PSM (Yaman et al., 2022).

Sheeba et al. (2014) studied the regulation of *hairy2* oscillations in the chick distal forelimb. When the Apical Ectodermal Ridge (AER) or the Zone of Polarizing Activity (ZPA), the key sources of FGF and Shh, respectively, were surgically removed from developing limbs, *hairy2* expression was abolished from the distal cyclic domain. This suggested that the EC could represent a functional intersection of these key molecules for limb proximal-distal outgrowth and patterning.

### 5.1 Strategies for accelerating/delaying the Embryo Clock

As described above, most attempts to modify the EC led to a complete disruption of the oscillations. However, an increasing number of experimental approaches have succeeded in altering the pace of the EC, which is key to understanding how this biological oscillator is regulated and the functional significance of its temporal dynamics (Table 5).

Following the work of by Takashima et al. (2011), Harima and colleagues generated a mouse mutant lacking only the first two introns of the Hes7 gene. This resulted in Hes7 oscillations with an 11 min-faster periodicity than the WT mice. This shorter cycle did not abolish EC oscillations nor somite formation. As predicted by the Clock and Wavefront model, since the EC presented a faster pace, this culminated in more and smaller somites (Harima et al., 2013). Recently, a similar approach was used to modify the tempo of *Hes1* oscillations in NPCs (Ochi et al., 2020). Deleting all the introns of the mouse *Hes1* gene accelerated expression oscillations by 13.6 min. On the other hand, by substantially increasing the primary transcript length the authors obtained exactly the opposite result, delayed EC in 13.5 min (Ochi et al., 2020). These are powerful examples of how transcriptional delays of EC genes can be modulated to tinker gene expression dynamics.

In the EC negative feedback regulatory loop, HES proteins are imported to the nucleus and repress their own transcription. Repression is lifted upon protein degradation, allowing a new transcription cycle to begin. Hence, by changing protein stability the repressive time on the gene promoter also changes, culminating in an overall alteration in the *tempo* of the EC. Through replacement of a Lysine for an Arginine in position 14, Hirata and colleagues were able to increase the half-life of the HES7 protein by 10 min, which led to an increase of 10,2 min in the global pace of the Embryo Clock (Hirata et al., 2004). A similar approach was applied by Kim et al. (2011) by knocking-out Nrarp, a negative effector of Notch signalling. This approach, suggested to delay NICD turnover, extended the EC pace by 5 min and resulted in the formation of fewer and defective vertebrae (Kim et al., 2011). Wiedermann et al. (2015) also accomplished to delay EC oscillations by stabilising NICD in the chick embryo, further corroborating these findings.

A wealth of knowledge on EC pace manipulation has also been provided using the zebrafish model. The Oates lab produced mutants in genes belonging to the Notch signalling pathway that displayed slower EC oscillations (Herrgen et al., 2010; Schröter and Oates, 2010). Knock-out of *notch1a*, *mib1*, and *deltaD* slowed the EC pace by 7%, 19%, and 23%, respectively (Herrgen et al., 2010), and mutating *hes6* delayed the EC by 6.5% (Schröter and Oates, 2010). Finally, chemical inhibition of the notch signalling pathway using DAPT delayed the EC by 18% (Herrgen et al., 2010). Corresponding delays in somitogenesis periodicity and a reduced final somite number corroborated the importance of Delta-Notch coupled oscillations for timely embryo body segmentation. Accordingly, elevation of Delta-Notch signalling accelerated EC oscillations and somite formation (Liao et al., 2016). Liao et al. (2016) created fish lines with 7 (*Dover*) or 100 (*Damascus*) extra copies of *deltaD*. Only the Damascus mutant displayed alterations to the EC, where oscillations were 1.6 min (6.4%) faster than in the wild-type. This increased the number of trunk segments by 7,6% and, despite the dramatic overexpression of *deltaD*, segmentation defects were rarely observed (Liao et al., 2016).

Other intercellular communication pathways contribute to the proper timing of the Embryo Clock. Sonic hedgehog (Shh) was shown to participate in EC *tempo* regulation. By comparing chicken PSM explants cultured with and without a source of Shh (notochord tissue and/or SHH-expressing cells), Resende et al. (2010) showed that the absence of Shh significantly delayed both EC oscillations and somite formation. Absence of Shh signalling led to an 85% increase of the EC period, from 90 min to approximately 2 h and 45 min. Similar experimental approaches showed that Wnt pathway inhibition by CKI-7 extended the EC pace from 90 to 120 min (Gibb et al., 2009). Comparable results were further obtained in the mouse model. Here, both CKI-7 treatment and activation of Wnt signalling using LiCl delayed *Hes7* oscillations (González et al., 2013). Using PSM-like tissues induced from mouse ESC, Yoshioka-Kobayashi et al. (2020) performed a high throughput chemical library screening and identified multiple small compounds capable of altering the period of *Hes7* oscillations by up to 40 min (Yoshioka-Kobayashi et al., 2020). These included modulators of a wide range of cellular processes and signalling pathways and further characterization of these alterations will surely improve our knowledge on EC operation.

Recently, we showed that the fibronectin-integrin-ROCK-NM II signalling axis regulates EC dynamics in the chicken PSM. Importantly, inhibition of actomyosin-mediated contractility delayed the period of *hairy1* (*hes4*) oscillations from 90 to 120 min (Gomes de Almeida et al., 2022), unveiling a previously unappreciated biomechanical regulation of the EC periodicity.

## 6 Pressing questions and future perspectives

Great attention has classically been dedicated to studying the molecular mechanisms involved in correct spatial positioning of cells/tissues/organs during embryo development, while the dynamics of gene expression over time was an under-represented concern. The discovery of a molecular Embryo Clock underlying somite formation gave way to a dramatic shift in this trend. Since it was first described in 1997, the EC has been characterized in multiple vertebrate species, evidencing a phylogenetically conserved mechanism. However, there are two aspects that differ depending on the organism: the pace of the EC and the specific oscillatory genes, although common signalling pathways are involved. The EC biological function has been tightly correlated with the segmentation of paraxial mesoderm, and mutations in Human EC genes give rise to severe congenital malformations of the axial skeleton, such as the phenotypes associated with spondylocostal dysostosis (Sparrow et al., 2012; Nobrega et al., 2021).

There is great interest in clarifying the EC clock dynamics and regulatory mechanisms in tissues other than the paraxial mesoderm and in different species, since this should help evidence what constitutes the central mechanism(s) of the clock, and which components are species/tissue-specific. Hairy-enhancer-of-split oscillatory expression is conserved in all species and tissues analysed, which has suggested their role as “core” members of the EC, but conclusive evidence for such fundamental clock components remains elusive. Studies on the mechanism(s) associated with the onset of gene expression oscillations during development might help elucidate whether EC operation is the output of a limited set of “core” clock genes or if it is an emergent property of the developing biological system, reverberating the oscillatory nature of the very first events during fertilization (e.g., Ca^2+^ oscillations induced upon sperm-oocyte fusion).

For many years, only *hairy2* was described to have cyclic expression in the chicken limb bud. More recently, Bhat et al. (2019) reported oscillations of *hes4* expression in chick limb micromass cultures. Here, *hes4* oscillates with a 6 h periodicity (Bhat et al., 2019), which matches the rate of limb *hairy2* oscillations *in vivo* (Pascoal et al., 2007). This suggests that the expression dynamics of both *hairy2* and *hes4* may be regulated by common mechanisms in the developing limb, further reinforcing the existence an EC-like mechanism operating during limb development (Sheeba et al., 2016). However, it is still unknown if this is conserved in other vertebrates and if altering *hairy2* or *hes4* expression may impact limb outgrowth and patterning. Clues arise from recent work evidencing that *Hes1* is a critical downstream effector of the Shh/Gli3 pathway in mouse limb development, where it regulates mesenchymal cell proliferation (Sharma et al., 2021). Importantly, *Hes1* overexpression promoted supernumerary digit formation and the authors concluded that *Hes1* regulates anterior boundary formation for digit development. Together, these studies suggest that synchronized *Hes* oscillations in the distal limb field could be functioning to prepattern the tissue for segment (digit) formation, which is reminiscent of the EC function in the PSM. Hence, the developing limb bud represents an additional extraordinary model system to further study EC regulation and function.

Despite the effort put into characterizing the EC, many fundamental questions remain unanswered. What triggers the onset of EC oscillations? What sets the *tempo* of the clock? What is the functional relevance of EC oscillations in different cell types and embryonic tissues? Is there a core component common to all vertebrates? Answering these and other pressing questions would allow us to understand how TIME is set and perceived for pattern formation during embryo development. Currently, there is a growing number of researchers employing novel experimental *in vitro* model systems that bring great promise to dissecting the EC mechanism in ways that have been hindered *in vivo* (reviewed in Diaz-Cuadros and Pourquié, 2021). These include the recently described Human *somitoids* derived from induced pluripotent stem cells (iPSC), which display segment formation and *Hes7* gene expression oscillations with the same periodicity as that previously describe for Human somitogenesis: ∼5 h (Sanaki-Matsumiya et al., 2022). After 25 years since the somitogenesis Embryo Clock was first described, the scientific community is more aware than ever of the existing knowledge gaps, but is also more equipped than ever to tackle the challenges ahead.
